# Spatial associations between esophageal lesions and surrounding tissues in esophageal fistula: a CAM-guided radiomics study

**DOI:** 10.3389/fonc.2026.1825313

**Published:** 2026-07-15

**Authors:** Ang Li, Lili Lin, Zewen Han, Jianqiang Ye, Junqing Lin, Han Jiang

**Affiliations:** 1 PET Center, Fujian Medical University Union Hospital, Fuzhou, China; 2 Clinical Research Center for Radiology and Radiotherapy of Fujian Province (Digestive, Hematological and Breast Malignancies), Fuzhou, China; 3 Fujian Key Laboratory of Intelligent Imaging and Precision Radiotherapy for Tumors (Fujian Medical University), Fuzhou, China; 4 Department of Interventional Radiology, Fujian Medical University Union Hospital, Fuzhou, China

**Keywords:** class activation mapping, deep learning, esophageal fistula, radiomics, risk stratification

## Abstract

**Objective:**

Esophageal fistula is a life-threatening complication following treatment of esophageal squamous cell carcinoma (ESCC). Early identification of high-risk patients remains challenging due to the limited predictive value of conventional imaging markers. This study aimed to develop and validate a Grad-CAM–guided deep learning–radiomics framework for risk prediction and spatial biomarker discovery using pre-treatment CT.

**Methods:**

In this retrospective study, 73 ESCC patients treated between January 2019 and December 2022 were included, of whom 25 (34.2%) developed esophageal fistula within one year. A 3D convolutional neural network (3D-CNN) was trained using stratified five-fold cross-validation. Grad-CAM was applied to localize discriminative regions, guiding radiomics feature extraction from the bilateral lungs, thoracic spine, and esophageal tumor. Radscores were constructed using least absolute shrinkage and selection operator regression. Logistic regression analyses were performed to identify independent predictors.

**Results:**

The 3D-CNN demonstrated stable performance across folds, achieving mean accuracy, sensitivity, and AUC of 0.809, 0.873, and 0.848, respectively. Grad-CAM revealed prominent activation differences in the lungs and thoracic spine rather than the central mediastinum. Radiomics analysis confirmed significant textural differences in all three regions (P < 0.001), with higher Radscores observed in the fistula group. In conventional multivariable logistic regression, Lung Radscore (OR = 11.55, P = 0.038) and Esophageal Tumor Radscore (OR = 192.3, P = 0.040) were associated with esophageal fistula, whereas conventional CT parameters lost statistical significance. Firth penalized logistic regression yielded more conservative estimates, with Esophageal Tumor Radscore remaining significant and Lung Radscore showing a borderline association.

**Conclusion:**

The Grad-CAM–guided 3D-CNN radiomics framework identified tumor-related and extratumoral imaging patterns associated with esophageal fistula, providing exploratory spatial biomarkers that warrant further validation.

## Graphical Abstract

**Figure:**
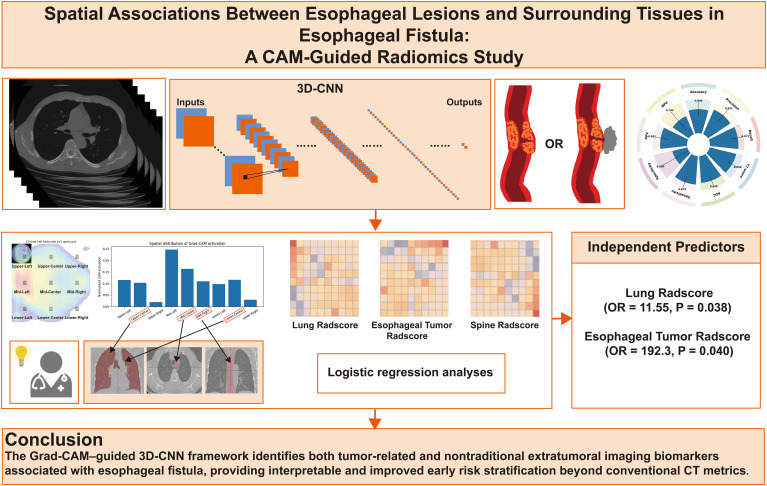

## 1 Introduction

Esophageal cancer is a common malignancy worldwide, with a high incidence and mortality rate (1). In China, esophageal cancer remains a significant public health burden, imposing substantial physical, psychological, and economic stress on affected patients and their families (2). Histologically, esophageal cancer is primarily classified into squamous cell carcinoma and adenocarcinoma. Treatment strategies typically include radiotherapy, chemotherapy, immunotherapy, and surgical resection, with modality selection guided by tumor stage and individual patient characteristics (3). In recent years, the advent of multidisciplinary and integrated treatment approaches-such as chemoradiotherapy followed by surgery-has markedly improved overall survival and quality of life in patients with esophageal cancer (4). However, treatment-related complications remain frequent and clinically consequential, including pulmonary infections, hemorrhage, and most notably, esophageal fistula (5). Among these, esophageal fistula is one of the most serious complications, often portending a poor prognosis (6, 7).

Currently, the diagnosis of esophageal fistula relies largely on imaging modalities such as CT and contrast esophagography. CT plays a pivotal role in identifying hallmark features of fistulas, including their size, location, and spatial relationship to adjacent structures (8). Despite these capabilities, conventional CT interpretation remains limited by subjectivity and reliance on radiologist expertise. Quantitative and objective tools for early risk prediction of fistula formation are lacking, especially in asymptomatic or preclinical stages.

Deep learning techniques, particularly convolutional neural networks (CNNs), have shown substantial promise in medical image analysis (9). CNNs can automatically extract and learn discriminative features from imaging data, often surpassing human-level performance in diagnostic classification tasks. Recent studies have demonstrated the feasibility of applying deep learning models to predict esophageal fistula based on CT images, offering improved accuracy and earlier detection (10, 11). However, a critical limitation of such models lies in their “black-box” nature—decisions are often difficult to interpret and lack clinical transparency.

Class Activation Mapping (CAM) is an emerging interpretability technique that highlights spatial regions within an input image that contribute most significantly to a model’s decision-making process (12). By generating intuitive visual overlays, CAM aids in revealing the “reasoning” behind CNN predictions and facilitates clinical trust in algorithmic outputs (13). Since its initial development, CAM has evolved into multiple variants—such as Grad-CAM, Adaptive-CAM, Score-CAM, and LayerCAM—each offering enhanced localization precision and model-agnostic applicability (14, 15). These tools provide a critical bridge between model performance and clinical explainability, thereby supporting informed decision-making in real-world applications. By highlighting image regions that most strongly influence model predictions, CAM enables clinicians to visualize and verify the model’s focus, fostering trust and aiding in clinical integration (16, 17). Importantly, CAM-guided regions can also be used to define biologically relevant regions of interest (ROIs) for further analysis, such as radiomics.

Radiomics allows for the extraction of high-dimensional, quantitative features from medical images, characterizing tissue heterogeneity and morphology beyond human visual assessment (18, 19). Radiomics-based workflows have been increasingly applied in medical image analysis for disease characterization, prognostic prediction, and treatment response assessment, and recent studies have further demonstrated the potential value of radiomics and transfer-learning strategies in imaging-based outcome prediction (20, 21). When combined with CAM-based localization, radiomics can be applied to automatically identified, model-informed ROIs, potentially uncovering subtle risk patterns not evident on conventional imaging.

In this study, we present an integrated framework that applies a deep learning model to predict the occurrence of esophageal fistula based on pre-treatment CT images, and then uses CAM-guided radiomics to quantify and interpret region-specific imaging biomarkers associated with fistula risk in patients with esophageal squamous cell carcinoma prior to treatment. Using CAM-based interpretability, we aim to visualize model attention and explore whether CNNs can identify potential risk regions that may fall outside conventional radiologic expectations. Ultimately, this approach seeks to facilitate early risk stratification and guide timely clinical interventions for high-risk individuals.

## 2 Materials and methods

### 2.1 Study population

This study was conducted in accordance with the principles stated in the Declaration of Helsinki, and it was approved by the institutional research ethics committee. The requirement for informed consent was waived (IRB No. 2025KY301) due to the nature of retrospective study. Consecutive patients with pathologically confirmed esophageal squamous cell carcinoma who underwent chest CT prior to treatment between January 2019 and December 2022 were screened from the institutional PACS system.

Patients meeting the following criteria were included: (a) histologically confirmed diagnosis of esophageal squamous cell carcinoma; (b) availability of baseline high-resolution chest CT scans of sufficient quality; (c) receipt of radiotherapy, chemotherapy, or immunotherapy; and (d) availability of clinical and imaging follow-up for at least 12 months. The exclusion criteria were as follows: (a) histologic subtype other than squamous cell carcinoma; (b) prior treatment or recurrent disease at the time of CT; (c) follow-up duration of less than one year; (d) history of concurrent or prior thoracic malignancies; (e) history of esophagectomy or other major esophageal surgery before baseline imaging; and (f) presence of esophageal fistula before treatment.

The study endpoint was defined as newly developed imaging-confirmed esophageal fistula occurring during treatment or within one year after treatment initiation. Diagnosis was based on CT and/or contrast esophagography. All included fistula events occurred without obvious tumor progression on imaging evaluation.

### 2.2 Imaging protocol

High-resolution chest CT scans were obtained in both supine and prone positions during full inspiration, without the use of contrast medium. The CT acquisition parameters included a tube voltage of 100 kVp, a tube current of 150–250 mAs, and a collimation width of 0.75 mm. All images were reconstructed in both axial planes with a section thickness of 1.0 mm, using a high-spatial-frequency reconstruction algorithm. All scans were obtained according to a standardized institutional high-resolution chest CT protocol, with consistent acquisition and reconstruction parameters across patients.

### 2.3 Deep learning model for predicting esophageal fistula

#### 2.3.1 Data processing

To ensure robust model evaluation while minimizing data leakage, a nested cross-validation framework was implemented. Specifically, patients were partitioned using stratified five-fold cross-validation, where in each fold approximately 80% of the data were used for model development and 20% served as an independent test set. Class proportions were preserved across folds.

Within each training fold, the model-development data were first split into an internal training subset and validation subset at a ratio of 80:20 using stratified sampling, resulting in an approximate overall ratio of 64% training, 16% validation, and 20% testing per fold. Class imbalance was addressed exclusively within the internal training subset by augmentation-based oversampling of the fistula class. Specifically, fistula-class CT volumes in the internal training subset were augmented until the class distribution was approximately balanced. The validation and test subsets were kept unchanged and were not used for oversampling, data augmentation, model training, hyperparameter optimization, or model selection; the test subset was used only for final performance evaluation. This procedure ensured that augmented samples were generated only from the model-training data and prevented information leakage into validation or test data.

Data augmentation strategies—including random flipping, brightness shifts, rotation, and zooming—were applied to the training subset to improve generalization and mitigate overfitting.

#### 2.3.2 Deep learning

A three-dimensional convolutional neural network (3D-CNN) was developed to classify patients as fistula-positive or fistula-negative based on pre-treatment CT volumes, which were resampled to 128 × 128 × 128 voxels. The network comprised an initial 3 × 3 × 3 convolutional layer with ReLU activation and batch normalization, followed by three residual blocks with progressive channel expansion (64, 128, and 256) and 2 × 2 × 2 max-pooling. Global average pooling and a fully connected layer with 128 neurons and 50% dropout were applied before a sigmoid output layer for binary classification.

The model was trained for up to 300 epochs with a batch size of 2 using binary cross-entropy loss and the Adam optimizer (initial learning rate 0.0001). Early stopping and ReduceLROnPlateau were employed to prevent overfitting, and the model with the lowest validation loss was retained for evaluation.

#### 2.3.3 Model performance analysis

Model performance was assessed using accuracy, sensitivity, specificity, precision, recall, F1-score, PPV, NPV and the area under the receiver operating characteristic curve (AUC).

### 2.4 CAM-guided radiomics

#### 2.4.1 Class activation mapping–guided ROI localization

CAM-guided ROI derivation and subsequent radiomics analyses were performed after 3D-CNN model evaluation as *post hoc* exploratory analyses and were not used for model training or cross-validation performance estimation.

To identify spatial regions most relevant to model decision-making, class activation mapping was performed using the Grad-CAM technique implemented in TensorFlow. A customized three-dimensional Grad-CAM pipeline was developed to accommodate volumetric CT inputs. Feature maps were extracted from the final convolutional layer of the second residual block, and gradients of the predicted class score with respect to these feature maps were computed. Channel-wise importance weights were obtained through global average pooling of gradients across spatial dimensions, followed by weighted summation of feature maps. The resulting activation map was passed through a rectified linear unit (ReLU) function and normalized to generate a 3D Grad-CAM heatmap.

To enable quantitative spatial analysis, each volumetric Grad-CAM map was projected along the axial dimension using maximum intensity projection, producing a two-dimensional activation map. The projected heatmap was subsequently partitioned into a standardized 3 × 3 spatial grid. Within each grid region, normalized activation intensity was calculated by summing high-response voxels exceeding a predefined threshold and dividing by total activation.

Group-level spatial activation patterns were obtained by averaging grid-wise activation values separately for patients with esophageal fistula and control subjects. Regions demonstrating the largest intergroup activation differences were identified as candidate imaging targets. These regions were then mapped back to corresponding anatomical structures on CT images and used to define regions of interest for downstream radiomics feature extraction.

To further assess whether CAM-highlighted regions contributed to model predictions, a region-masking analysis was performed. Based on the predefined 3 × 3 grid, regions 2, 8, and 6 corresponded to the bilateral lung- and thoracic spine-related areas. Within these regions, the top 50% CAM-activated voxels were replaced by the mean CT intensity of the corresponding volume, and the masked images were re-entered into the trained model. Predicted probabilities before and after masking were compared using the Wilcoxon signed-rank test.

#### 2.4.2 Regions of interest segmentation

ROIs were segmented within the predefined anatomical structures using a hybrid delineation strategy. Manual segmentation was performed in 3D Slicer (version 5.8.0), complemented by a semi-automated region-growing algorithm implemented in Insight Segmentation and Registration Toolkit (version 3.8) to improve boundary consistency and reduce operator variability.

Segmentation was conducted in three dimensions with careful boundary verification to ensure accurate representation of anatomical morphology while avoiding inclusion of adjacent tissues. Quality control procedures included visual inspection and boundary refinement, and only ROIs meeting predefined consistency criteria were retained for subsequent radiomics feature extraction. Segmentation reproducibility was assessed in a subset of ROIs by two observers using intraclass correlation coefficients (ICCs) of extracted radiomics features; ICC > 0.80 was considered acceptable.

#### 2.4.3 Radiomics feature extraction

Radiomics features were extracted using PyRadiomics (version 3.0.1), which adheres to the Image Biomarker Standardization Initiative (IBSI) guidelines. Prior to feature extraction, all ROIs were resampled to isotropic voxel spacing of 1 × 1 × 1 mm³ using B-spline interpolation. Gray-level discretization was performed using a fixed bin width of 25 Hounsfield units.

To enhance feature representation, multiple image filters were applied, including Laplacian-of-Gaussian (LoG) filtering with sigma = 1 mm and wavelet decompositions. A comprehensive set of radiomics features was extracted from both original and filtered images, including first-order statistics, shape descriptors, and texture features derived from gray-level co-occurrence matrix (GLCM), gray-level run length matrix (GLRLM), gray-level size zone matrix (GLSZM), gray-level dependence matrix (GLDM), and neighboring gray-tone difference matrix (NGTDM).

#### 2.4.4 Feature selection and radiomics scores

Feature selection and Radiomics Scores was performed using the least absolute shrinkage and selection operator (LASSO) regression implemented in the glmnet R package.

### 2.5 Risk stratification for esophageal fistula

Both conventional CT parameters—such as esophageal wall thickness, maximum luminal diameter, tumor invasion depth, and osteophyte count—and CAM-guided Radscores were analyzed as potential risk factors for fistula formation.

Univariate logistic regression was conducted to assess the association between each variable and the occurrence of esophageal fistula, with a P value < 0.05 considered statistically significant. All variables were then entered into a multivariate logistic regression model using the enter method to identify independent predictors. In the multivariate analysis, statistical significance was also defined as P < 0.05. Odds ratios and corresponding 95% confidence intervals were reported for all models. Multicollinearity among predictors was assessed using generalized variance inflation factors (GVIFs), with adjusted GVIF values below 5 considered to indicate no severe multicollinearity. Given the limited number of fistula events and the possibility of sparse-event bias, Firth penalized multivariable logistic regression was additionally performed as a sensitivity analysis. Calibration of the radiomics-based multivariable logistic regression model was assessed using a calibration curve comparing predicted probabilities with observed frequencies of esophageal fistula. The Brier score was calculated to quantify overall prediction error.

### 2.6 Statistical analysis

Univariate analysis was performed using the independent-samples t-test or Mann–Whitney U test for continuous variables and the chi-square test for categorical variables. A two-sided P value < 0.05 was considered statistically significant. For radiomics feature comparisons, Benjamini–Hochberg false discovery rate correction was applied, and FDR-adjusted P values were reported. Statistical analyses were conducted using R software (version 4.4.3; R Foundation for Statistical Computing) and Python (version 3.7; Python Software Foundation). Radiomics feature extraction was performed using PyRadiomics (version 3.0.1).

## 3 Results

### 3.1 Study cohort

**Figure 1:**
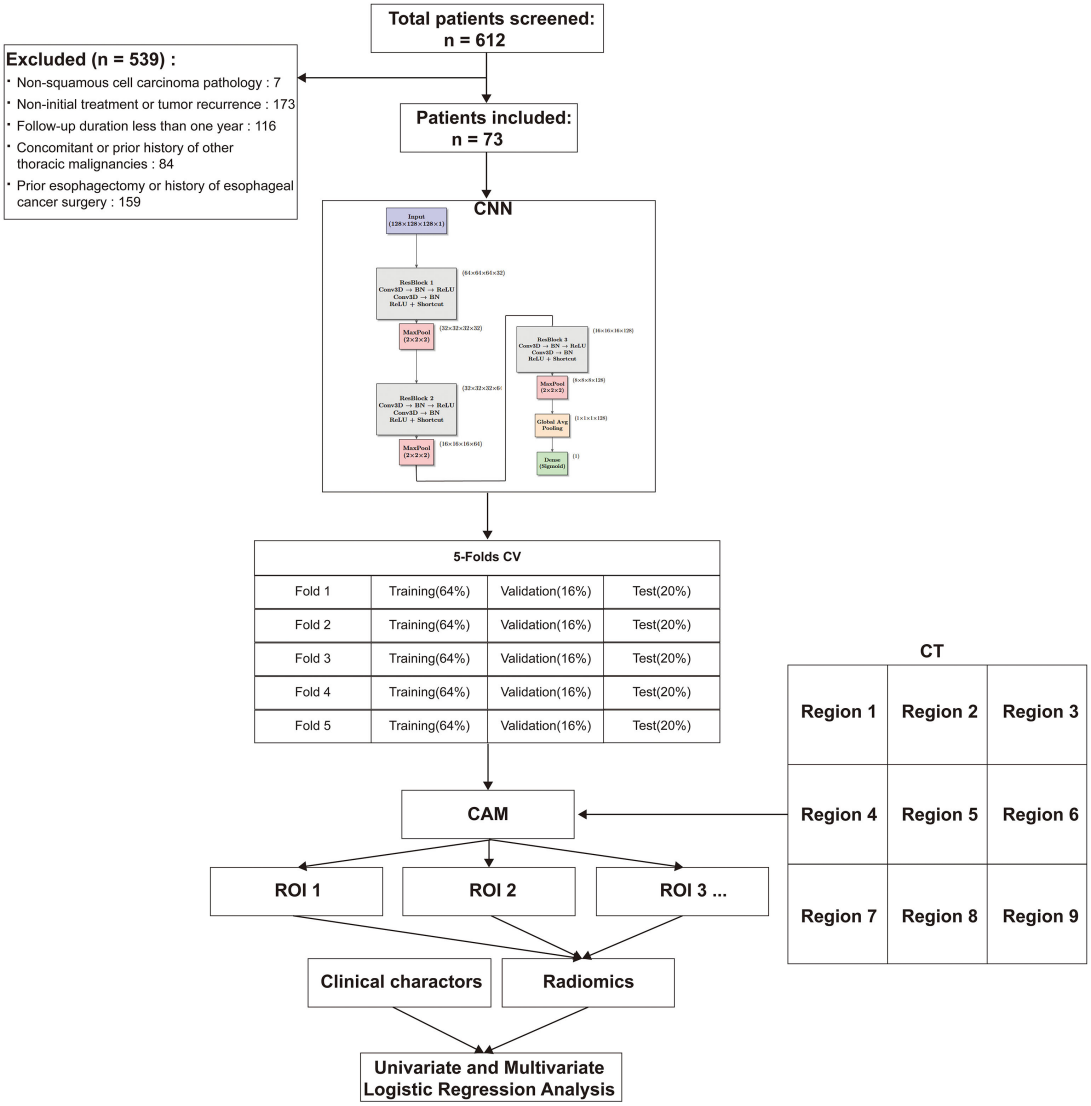
Flowchart of patient inclusion and study workflow, illustrating model development and the CAM-guided radiomics framework for fistula risk prediction.

A total of 73 patients met the inclusion criteria and were included in the final analysis (**Figure 1**). Among these, 25 patients (34.2%) developed esophageal fistula within one year following treatment, while 48 patients (65.8%) did not. Baseline characteristics and imaging features are summarized in **Table 1**. There were no statistically significant differences between the fistula and control groups in terms of age (61.6 ± 9.7 vs. 62.8 ± 10.2 years, *P* = 0.633) or sex distribution (88.0% vs. 72.9% male, *P* = 0.238).

**Table 1:** Comparison of baseline demographics and imaging features between control and esophageal fistula groups.

Variable	Control group (n=48)	Fistula group (n=25)	P Value
Demographics			
Sex			0.238
Male	35 (72.9)	22 (88.0)	
Female	13 (27.1)	3 (12.0)	
Age (years)	62.8 ± 10.2	61.6 ± 9.7	0.633
Imaging features			
Esophageal wall thickness (mm)	17.6 ± 5.6	22.8 ± 5.6	< 0.001
Maximum lumen dilation diameter (mm)	9.2 (0-31.5)	14.7 (0-52.7)	0.042
Tumor invasion depth (mm)	7.0 (0-18.0)	7.1 (0-19.9)	0.524
Osteophyte count			0.672
<3	27 (56.3)	12 (48.0)	
≥3	21 (43.8)	13 (52.0)	
Tumor location			0.768
Upper esophagus	10 (20.8)	5 (20.0)	
Middle esophagus	25 (52.1)	15 (60.0)	
Lower esophagus	13 (27.1)	5 (20.0)	

Patients who developed fistulas demonstrated significantly greater esophageal wall thickness on baseline CT (22.8 ± 5.6 mm vs. 17.6 ± 5.6 mm, *P* < 0.001) and larger maximum luminal dilation (14.7 mm vs. 9.2 mm, *P* = 0.042). Tumor invasion depth, tumor location, and osteophyte count were not significantly different between groups (*P* > 0.05 for all comparisons).

### 3.2 Performance of the deep learning model

To obtain a robust estimation of model performance while minimizing sampling bias, a stratified five-fold cross-validation strategy was adopted, preserving the proportion of esophageal fistula cases within each fold.

**Figure 2:**
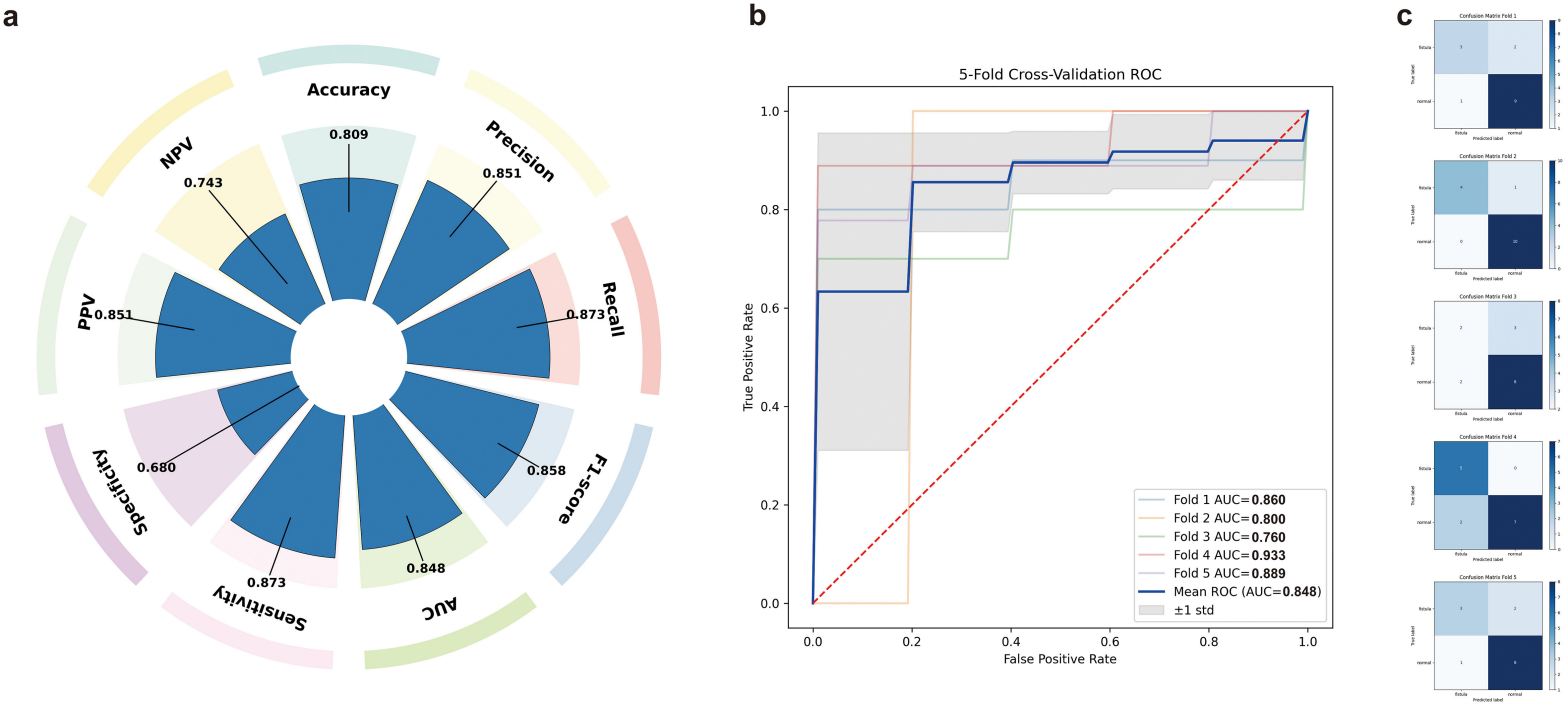
Performance evaluation of the 3D-CNN model. **(A)** Mean performance metrics from stratified five-fold cross-validation. **(B)** ROC curves across five folds. **(C)** Corresponding confusion matrices.

Across the five folds, the proposed 3D convolutional neural network demonstrated stable predictive performance. The mean accuracy, precision, recall, F1-score, and AUC were 0.809, 0.851, 0.873, 0.858, and 0.848, respectively. Sensitivity and specificity averaged 0.873 and 0.680, indicating strong detection capability for fistula occurrence with moderate specificity. The mean positive predictive value and negative predictive value were 0.851 and 0.743, respectively (**Figure 2**).

Performance variability across folds was limited, with accuracy ranging from 0.667 to 0.933 and AUC ranging from 0.760 to 0.933, demonstrating consistent discriminative ability of the model. Notably, sensitivity remained high across folds (range, 0.778–1.000), supporting the model’s robustness in identifying patients at risk for esophageal fistula (**Supplementary Table 1**).

### 3.3 Grad-CAM–based ROI identification

**Figure 3:**
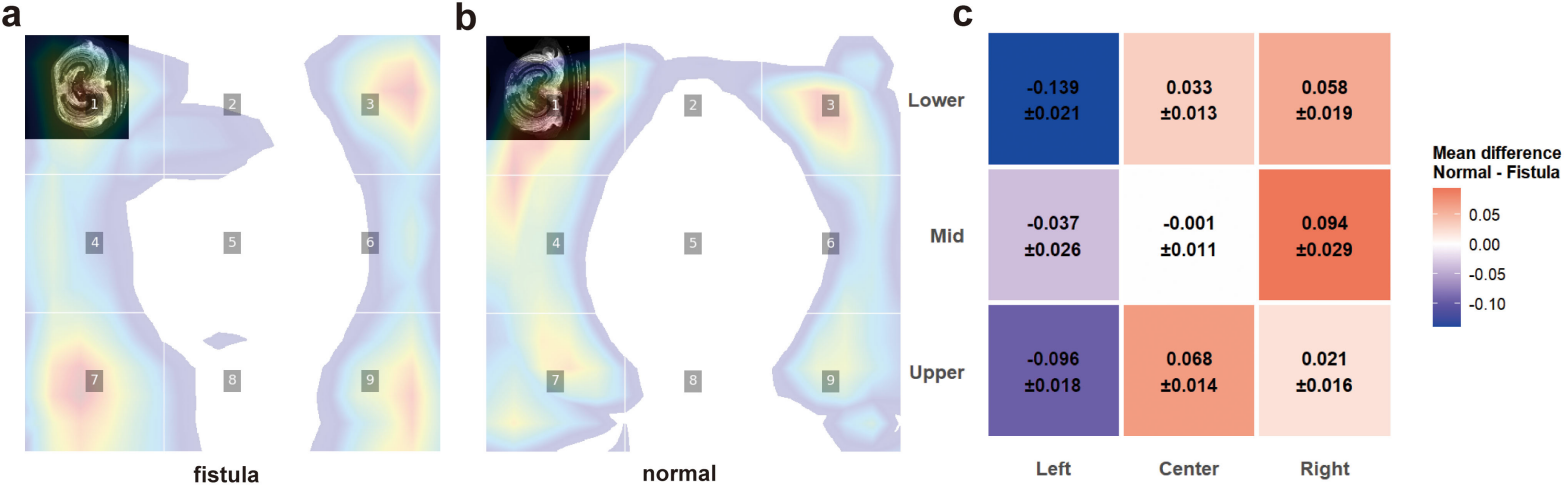
Grad-CAM visualization and intergroup spatial differences. **(A)** Representative heatmap from a patient with esophageal fistula. **(B)** Representative heatmap from a non-fistula patient. **(C)** Mean spatial attention difference matrix between groups. Values are presented as mean ± standard deviation of activation differences across patients.

Grad-CAM activation maps generated from the trained 3D-CNN were spatially summarized using a 3 × 3 grid across thoracic CT slices, and mean intergroup activation differences were calculated for each region (**Figure 3**). The four corner regions were excluded due to limited anatomical representation.

Greater activation differences were observed in the bilateral lungs and thoracic vertebrae, whereas comparatively smaller differences were noted in the central mediastinal region (**Figure 3**). Based on these results, CAM-guided regions of interest were defined for radiomics analysis, including the bilateral lungs, thoracic spine, and esophageal tumor.

To further evaluate whether CAM-highlighted regions contributed to model output, region-masking analysis was performed. Masking the top 50% CAM-activated voxels within grid regions 2, 8, and 6 reduced the median predicted probability of esophageal fistula from 0.073 to 0.014 in the overall cohort, with decreased probabilities in 70 of 73 patients (95.9%; Wilcoxon signed-rank test, P = 1.13 × 10^-^¹²). In the fistula group, the median probability decreased from 0.983 to 0.942, with reductions observed in all 25 patients (P = 2.98 × 10^-8^). In the non-fistula group, the median probability decreased from 0.030 to 0.009, with reductions observed in 45 of 48 patients (P = 2.04 × 10^-8^). These results indicate that CAM-activated voxels in these regions contributed to the model output. These results are shown in **Supplementary Figure 1**.

### 3.4 Radiomics analysis of CAM-guided ROIs

**Figure 4:**
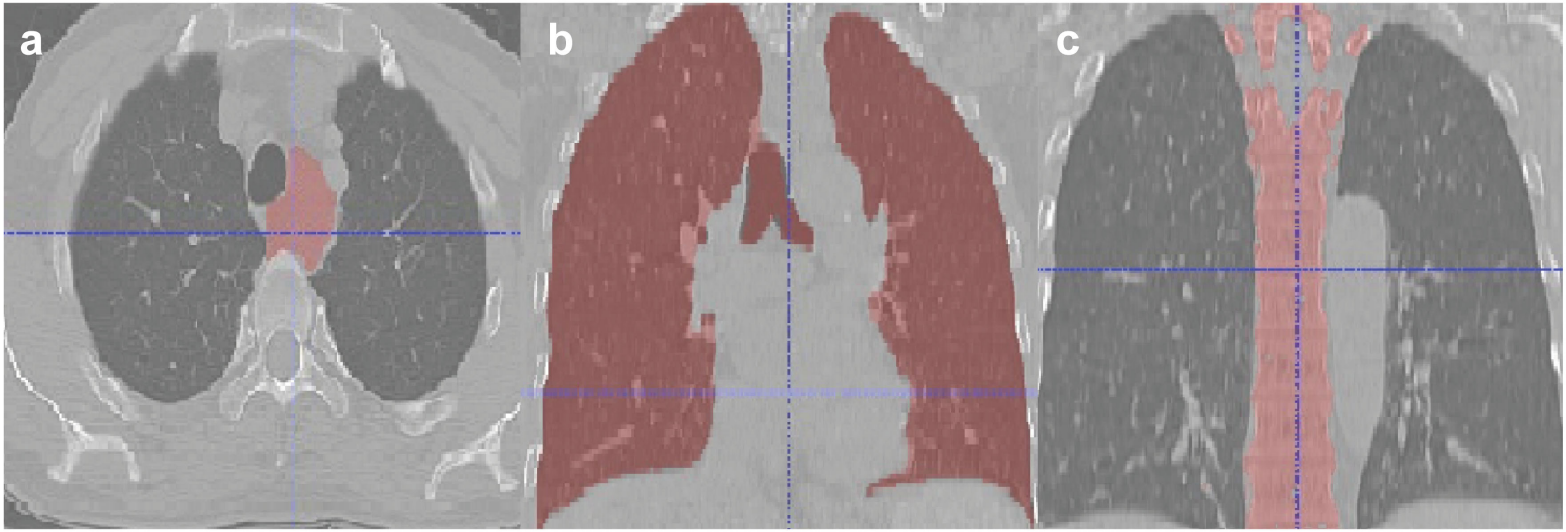
CAM-guided regions of interest (ROIs) for radiomics analysis. **(A)** Esophageal tumor ROI. **(B)** Bilateral lung ROIs. **(C)** Thoracic spine ROI.

Radiomics features were quantitatively extracted from three anatomically distinct, Grad-CAM–guided ROIs: the bilateral lungs, thoracic spine, and esophageal tumor (**Figure 4**). Comparative analysis revealed significant textural and intensity-based differences between patients who developed esophageal fistulas and those who did not. Most selected features remained significant after Benjamini–Hochberg FDR correction (**Table 2**). Reproducibility analysis showed acceptable feature robustness, with ICCs greater than 0.95 for lung and thoracic spine ROIs and greater than 0.80 for the esophageal tumor ROI (**Supplementary Table 2**).

**Table 2:** Comparison of radiomics features and Radscore between control and fistula groups across different ROIs.

Lung radiomics feature	Control group (n=48)	Fistula group (n=25)	P value	FDR-P value
Texture analysis				
GLCM IDMN (log-sigma=1 mm)	0.998 ± 0.0008	0.997 ± 0.0007	0.133	0.133
GLDM LargeDepLowGrayEmphasis (LLH)	0.0284 ± 0.0222	0.0604 ± 0.0480	0.004	0.009
GLSZM LowGrayZoneEmphasis (LLH)	0.00043 ± 0.00017	0.00057 ± 0.00025	0.013	0.018
NGTDM Contrast (HHL)	0.00302 ± 0.00206	0.00546 ± 0.00341	0.003	0.009
First-order statistics				
Median Intensity (HHH)	-0.0124 ± 0.0221	0.00976 ± 0.0568	0.071	0.080
Higher-order features				
GLCM MCC (HHH)	0.336 ± 0.106	0.281 ± 0.0745	0.011	0.018
GLDM DependenceVariance (HHH)	12.0 ± 3.81	9.64 ± 3.67	0.014	0.018
GLCM IMC2 (LLL)	0.857 ± 0.0521	0.807 ± 0.0712	0.003	0.009
GLCM MCC (LLL)	0.778 ± 0.0526	0.733 ± 0.0592	0.002	0.009
**Lung Radscore**	**0.235 ± 0.144**	**0.549 ± 0.160**	**<0.001**	
Spine radiomics feature				
First-order statistics				
Skewness (LLH)	-0.985 ± 0.429	-0.562 ± 0.574	0.002	0.004
Median Intensity (HLL)	-0.655 ± 2.75	2.15 ± 2.84	<0.001	0.001
Minimum Intensity (HHL)	-837 ± 613	-1420 ± 688	<0.001	0.002
GLCM features				
Correlation (LLH)	0.336 ± 0.094	0.404 ± 0.111	0.012	0.014
GLDM features				
Low Gray-Level Emphasis (LLH)	0.000549 ± 0.000303	0.000929± 0.000844	0.038	0.038
LargeDependenceLowGray-Level Emphasis (HLL)	0.00969 ± 0.00761	0.00400 ± 0.00584	<0.001	0.002
Dependence Variance (HHH)	14.8 ± 4.00	11.2 ± 5.84	0.008	0.011
Dependence Variance (LLL)	6.84 ± 4.47	3.12 ± 3.67	<0.001	0.001
**Spine Radscore**	**0.281 ± 0.117**	**0.460 ± 0.0985**	**<0.001**	
Esophagus radiomics feature				
First-order statistics				
Energy (log-sigma=1 mm)	1.26 × 10^8^ ± 8.78 × 10^7^	3.04 × 10^8^ ± 2.17 × 10^8^	<0.001	0.001
Total Energy (log-sigma=1 mm)	1.26 × 10^8^ ± 8.78 × 10^7^	3.04 × 10^8^ ± 2.17 × 10^8^	<0.001	0.001
Minimum Intensity (LHL)	-906 ± 532	-1890 ± 1320	0.001	0.002
Kurtosis (LHL)	20.6 ± 9.70	29.6 ± 17.7	0.024	0.032
Skewness (HHH)	-0.0163 ± 0.111	-0.101 ± 0.241	0.107	0.122
GLCM features				
Inverse Variance (LLH)	0.447 ± 0.031	0.415 ± 0.038	<0.001	0.001
Idn (LHL)	0.968 ± 0.009	0.972 ± 0.009	0.035	0.046
IMC1 (HHL)	-0.0595 ± 0.0139	-0.0699 ± 0.0225	0.042	0.051
MCC (HHL)	0.463 ± 0.073	0.516 ± 0.067	0.003	0.005
GLSZM features				
SmallAreaHighGray-Level Emphasis (log-sigma=1 mm)	196 ± 165	511 ± 551	0.009	0.013
Zone Entropy (log-sigma=1 mm)	6.70 ± 0.299	6.98 ± 0.239	<0.001	<0.001
Low Gray-Level Zone Emphasis (HLH)	0.0249 ± 0.0213	0.0460 ± 0.0713	0.159	0.159
GLRLM features				
Short Run Low Gray-Level Emphasis (LHL)	0.00135 ± 0.00098	0.000495 ± 0.00055	<0.001	<0.001
Run Length Non-Uniformity (HLL)	18000 ± 8160	36400 ± 17000	<0.001	<0.001
Low Gray-Level Run Emphasis (LLL)	0.000665 ± 0.00060	0.000265 ± 0.00014	<0.001	<0.001
Run Length Non-Uniformity (LLL)	20400 ± 7260	33900 ± 12900	<0.001	<0.001
GLDM features				
Dependence Non-Uniformity (HHL)	3140 ± 1660	7650 ± 4360	<0.001	<0.001
**Esophagus Radscore**	**0.180 ± 0.131**	**0.653 ± 0.223**	**<0.001**	

GLCM, gray-level co-occurrence matrix; GLDM, gray-level dependence matrix; GLSZM, gray-level size zone matrix; NGTDM, neighborhood gray-tone difference matrix; IDMN, inverse difference moment normalized; MCC, maximal correlation coefficient; IMC2; informational measure of correlation 2. log-sigma=1 mm/LLH/HLL/HHL/HHH/LLL represent different wavelet decomposition levels. Lung, Spine, and Esophagus Radscores were derived from radiomic features of the corresponding ROIs.

In the lung ROI, several higher-order texture features remained significant after FDR correction, including GLDM LargeDependenceLowGrayEmphasis (P = 0.004; FDR-P = 0.009), GLSZM LowGrayZoneEmphasis (P = 0.013; FDR-P = 0.018), and NGTDM Contrast (P = 0.003; FDR-P = 0.009), indicating predominance of low-intensity extensive zones and increased local heterogeneity. In the thoracic spine ROI, both first-order and texture features differed significantly. Skewness (P = 0.002) and GLDM DependenceVariance (P = 0.008) were higher in the fistula group, suggesting greater intensity heterogeneity and structural irregularity. The esophageal tumor ROI showed the most pronounced divergence. First-order features (Energy and Total Energy, both P < 0.001) and multiple texture features from GLCM and GLRLM domains were significantly elevated, reflecting increased microstructural complexity.

Radscores derived from each ROI were significantly higher in the fistula group than in controls (all P < 0.001). In the lung ROI, the mean Radscore was 0.549 ± 0.160 versus 0.235 ± 0.144. In the thoracic spine ROI, values were 0.460 ± 0.099 versus 0.281 ± 0.117. The esophageal tumor ROI showed the largest difference, with mean Radscores of 0.653 ± 0.223 in the fistula group and 0.180 ± 0.131 in controls (**Table 2**).

### 3.5 Factors associated with esophageal fistula

In univariate logistic regression, esophageal wall thickness (P = 0.002), maximum luminal diameter (P = 0.031), and all three regional Radscores (lung, thoracic spine, and esophageal tumor; all P < 0.001) were significantly associated with fistula occurrence. Age, sex, tumor invasion depth, osteophyte count, and tumor location were not significant.

In conventional multivariable logistic regression, the Lung Radscore (OR = 11.55; 95% CI: 1.61–244.23; P = 0.038) and Esophageal Tumor Radscore (OR = 192.30; 95% CI: 4.10–11311.60; P = 0.040) were associated with fistula occurrence, whereas conventional CT parameters were not significant (**Table 3**). Collinearity assessment showed that all adjusted GVIF values were below 5, suggesting no severe multicollinearity among the included predictors (**Supplementary Table 1**). Given the wide confidence intervals, Firth penalized multivariable logistic regression was performed as a sensitivity analysis (**Table 4**). After penalization, the Esophageal Tumor Radscore remained significantly associated with fistula occurrence (OR = 7.61; 95% CI: 1.17–365.95; P = 0.029), whereas the Lung Radscore showed a borderline association (OR = 3.67; 95% CI: 0.98–22.20; P = 0.054). These results suggest that CAM-guided Radscores may be associated with fistula occurrence, although the estimates should be interpreted cautiously because of the limited number of events. Calibration analysis of the radiomics-based multivariable logistic regression model showed the relationship between predicted probability and observed frequency of esophageal fistula, with a Brier score of 0.0530 (**Supplementary Figure 2**).

**Table 3:** Univariate and multivariate logistic regression analysis with radiomics score.

Variable	Univariate		Multivariate	
	OR (95% CI)	P Value	OR (95% CI)	P Value
Sex (Male)	2.72 (0.71 - 10.45)	0.150	1.68 (0.04 - 67.96)	0.804
Age	0.99 (0.95 - 1.03)	0.633	0.88 (0.71 - 1.10)	0.237
Esophageal wall thickness	1.18 (1.06 - 1.31)	0.002	1.12 (0.81 - 1.56)	0.459
Maximum lumen dilation diameter	1.08 (1.01 - 1.16)	0.031	1.14 (0.89 - 1.46)	0.287
Tumor invasion depth	1.04 (0.92 - 1.19)	0.492	1.03 (0.74 - 1.44)	0.879
Osteophyte count ≥3	1.39 (0.57 - 3.37)	0.503	7.21 (0.38 - 136.89)	0.233
Tumor location- Middle esophagus	1.20 (0.35 - 4.08)	0.775	0.09 (0.00 - 2.47)	0.274
Tumor location- Lower esophagus	0.77 (0.17 - 3.50)	0.730	0.08 (0.00 - 8.42)	0.354
Lung Radscore	26360.2 (440.1 - ∞)	<0.001	107649.5 (1.53 - ∞)	0.038
Spine Radscore	285332.3 (188.9 - ∞)	<0.001	0.00 (0.00 - ∞)	0.163
Esophagus Radscore	79850.7 (141.5 - ∞)	<0.001	680417.7 (1.56 - ∞)	0.040

**Table 4:** Conventional and firth penalized multivariable logistic regression analysis of factors associated with esophagomediastinal fistula.

Variable	Conventional logistic OR (95% CI)	P value	Firth logistic OR (95% CI)	P value
Sex (Male)	1.69 (0.03–264.98)	0.804	0.88 (0.08–18.12)	0.917
Age	0.88 (0.69–1.05)	0.236	0.97 (0.83–1.08)	0.573
Esophageal wall thickness	1.12 (0.83–1.63)	0.459	1.06 (0.86–1.35)	0.560
Maximum lumen dilation diameter	1.14 (0.92–1.51)	0.287	1.06 (0.91–1.29)	0.498
Tumor invasion depth	1.03 (0.75–1.57)	0.879	0.99 (0.79–1.34)	0.921
Osteophyte count ≥3	7.21 (0.33–299.49)	0.233	2.30 (0.26–30.60)	0.453
Tumor Location- top esophagus	0.09 (0.00–6.86)	0.274	0.33 (0.01–6.59)	0.478
Tumor Location- Lower esophagus	0.08 (0.00–14.15)	0.354	0.39 (0.01–13.69)	0.595
Lung Radscore	11.55 (1.61–244.23)	0.038	3.67 (0.98–22.20)	0.054
Spine Radscore	0.07 (0.00–1.64)	0.163	0.44 (0.02–2.84)	0.433
Esophagus Radscore	192.30 (4.10–11311.60)	0.040	7.61 (1.17–365.95)	0.029

## 4 Discussion

In this study, we developed a 3D convolutional neural network based on pre-treatment CT to predict esophageal fistula risk in patients with esophageal squamous cell carcinoma. Using stratified five-fold cross-validation, the model demonstrated stable performance, with a mean AUC of 0.848, high sensitivity (0.873), and moderate specificity (0.680), supporting its potential utility for early risk screening.

The 3D-CNN in this study was used primarily as an imaging feature discovery and interpretability tool rather than as a stand-alone clinical prediction model. Given the limited cohort size, the cross-validation performance should be interpreted as an internal feasibility estimate rather than evidence of generalizable clinical performance. The main value of this framework lies in its ability to generate Grad-CAM maps and identify candidate anatomical regions for downstream radiomics analysis. Thus, this study should be considered an exploratory investigation of spatial imaging biomarkers associated with esophageal fistula.

Notably, Grad-CAM analysis showed that model attention was predominantly focused on the bilateral lungs and thoracic vertebrae rather than solely on the esophageal wall. Quantitative activation differences further confirmed greater intergroup variation in these surrounding thoracic structures compared with the central mediastinum. This finding suggests that model-derived imaging patterns associated with fistula occurrence may extend beyond the primary tumor region, although their biological significance requires further validation. Radiomics features extracted from these CAM-guided regions were significantly associated with fistula occurrence and remained independent predictors in multivariate analysis, indicating that deep learning–driven spatial attention can facilitate identification of clinically meaningful imaging biomarkers beyond traditional human-defined regions.

Although the region-masking analysis supported the contribution of CAM-activated lung- and spine-related voxels to model predictions, Grad-CAM and masking analyses cannot establish biological causality. Activation patterns may be influenced by body habitus, respiratory status, lung inflation, scan positioning, motion artifacts, age-related vertebral changes, or acquisition-related variability. Therefore, the lung and thoracic spine findings should be interpreted as model-relevant spatial imaging information rather than direct mechanistic evidence.

Prior studies investigating risk factors for esophageal fistula have primarily focused on macroscopic imaging markers, such as tumor location, maximal esophageal wall thickness, or degree of luminal expansion (22, 23). While some of these features were statistically significant in univariate analysis in our cohort, they did not retain statistical significance in multivariate modeling when CAM-guided Radscores were included. This finding suggests that conventional morphologic criteria may not fully capture imaging heterogeneity associated with fistula occurrence. A recent study developed a radiomics-clinical nomogram that combined CT-derived tumor texture features and clinical indicators to predict esophageal fistula, achieving an AUC of 0.867 in the validation cohort; however, the model was based solely on manually segmented primary tumor regions and did not consider extra-esophageal structures or provide visual interpretability (24). Unlike prediction-oriented radiomics or deep learning models, the present study used 3D-CNN and Grad-CAM primarily as an imaging feature discovery tool to explore CT-derived spatial factors beyond conventional clinical and radiologic features. In contrast, our integrative approach combines volumetric 3D-CNN modeling with CAM-based localization and quantitative radiomics analysis, enabling exploratory identification of spatially distributed imaging biomarkers. The wide confidence intervals in conventional logistic regression suggest statistical uncertainty and possible sparse-event bias. Although Firth penalized regression attenuated the effect estimates, the overall direction of association for the Lung Radscore and Esophageal Tumor Radscore was generally consistent, supporting their exploratory relevance while underscoring the need for validation in larger cohorts.

The observation that the deep learning model primarily focused on the lung parenchyma and thoracic spine—rather than the esophageal tumor itself—may warrant further investigation into possible biological and treatment-related explanations. From a molecular standpoint, both radiotherapy and chemotherapy can induce tissue-level changes that compromise structural integrity and healing capacity. Radiation is known to initiate endothelial senescence, disrupt microvascular integrity, and activate TGF-β–mediated fibroblast differentiation, promoting localized fibrosis and impaired regeneration (25, 26). Similarly, chemotherapeutic agents such as bleomycin and paclitaxel trigger fibrotic remodeling via activation of myofibroblasts, increased ECM deposition, and reactive oxygen species accumulation, often through the TGF-β/Smad signaling axis (27, 28). These processes result in increased tissue stiffness and heterogeneity, which may predispose the periesophageal lung tissue to microinjury, inflammation, or secondary infection. The periesophageal lung parenchyma may be vulnerable to radiation- or inflammation-related alterations in aeration, perfusion, and interstitial structure, which could be reflected by local CT texture heterogeneity.

Additionally, chemotherapy-related immune activation and TRP channel–mediated mechanotransduction have been implicated in amplifying inflammatory-fibrotic cascades, further destabilizing adjacent structures (29, 30). In parallel, radiation to the vertebral body may compromise bone biomechanics, while chronic osteophyte formation may exert mechanical pressure on a structurally weakened posterior esophageal wall (31, 32). Thoracic spine activation may therefore reflect biomechanical, degenerative, or treatment-related changes near the posterior esophageal boundary. Such biomechanical interactions have been supported by mechanics-informed disease models and are consistent with the pathogenesis of delayed fistula formation in patients with localized tissue damage and impaired wound healing capacity.

These biologically plausible processes align with our radiomics analysis, which revealed significant alterations in texture features—such as gray-level non-uniformity and entropy—within the CAM-highlighted pulmonary and spinal regions. Prior studies have demonstrated that these features are sensitive to subclinical changes in tissue composition, fibrosis, and perfusion abnormalities (33, 34). It is thus likely that the deep learning model captured spatially distributed, therapy-induced vulnerabilities that fall outside routine radiologic inspection. Moreover, explainable AI frameworks—such as ELNet and multi-task networks—have shown that incorporating regional and contextual features enhances predictive accuracy and reveals previously underappreciated risk patterns (35, 36). Taken together, these findings indicate that the CAM-highlighted regions contributed to model output; however, they should be interpreted as exploratory spatial imaging patterns rather than causal evidence of treatment-related vulnerability or microenvironmental pathology.

The potential clinical relevance of our findings should be interpreted cautiously. Esophageal fistula is a severe complication, and current risk assessment still relies largely on clinical judgment and conventional imaging interpretation. The CAM-guided framework provides exploratory CT-based spatial imaging information that may inform future studies on fistula risk assessment beyond tumor morphology alone. However, it is not intended for immediate clinical deployment or direct individualized intervention. Larger prospective multicenter studies are required to determine its clinical utility.

This study has several limitations. First, this was a retrospective single-center study with only 73 patients and 25 fistula events. Given the small cohort and use of a volumetric 3D-CNN, overfitting cannot be excluded, and the reported performance should be viewed as an internal feasibility estimate rather than evidence of generalizable clinical performance. External validation in larger multi-institutional cohorts is required. Second, although CAM enhanced model interpretability, it provides relatively coarse localization; attention patterns may also be affected by anatomical, physiological, or acquisition-related confounders. More advanced explainability techniques, together with occlusion testing, region masking, and activation-map reproducibility assessment, may improve spatial precision in future work. Third, this study focused on CT-derived spatial imaging factors and did not comprehensively incorporate clinical or treatment-related predictors. Future studies should integrate multimodal imaging, clinical, and treatment-related variables to improve risk assessment. Finally, histopathologic correlation for the lung and vertebral regions was unavailable, limiting mechanistic interpretation. Future studies should focus on prospective validation, evaluation of clinical impact, and extension of this framework to other treatment-related complications in thoracic oncology.

## 5 Conclusion

We developed an explainable 3D-CNN, Grad-CAM, and radiomics framework to explore CT-based imaging biomarkers associated with esophageal fistula in patients with esophageal squamous cell carcinoma. The 3D-CNN served primarily as a spatial feature discovery tool, enabling Grad-CAM–guided localization of candidate risk regions for radiomics analysis. The Lung Radscore and Esophageal Tumor Radscore were independently associated with fistula occurrence, suggesting that both tumor-related and extratumoral imaging features may contribute to fistula risk beyond conventional CT morphology. Further multicenter validation is required.

## Data Availability

The raw data supporting the conclusions of this article will be made available by the authors, without undue reservation.

## References

[1] Bray F Laversanne M Sung H Ferlay J Siegel RL Soerjomataram I . Global cancer statistics 2022: GLOBOCAN estimates of incidence and mortality worldwide for 36 cancers in 185 countries. CA Cancer J Clin. (2024) 74(3):229–63. doi: 10.3322/caac.21834 38572751

[2] Chen X Zheng R Xu X Wang Z Huang G Wu R . Frailty and health-related quality of life in elderly patients undergoing esophageal cancer surgery: a longitudinal study. Asian Nurs Res (Korean Soc Nurs Sci). (2024) 18(2):125–33. doi: 10.1016/j.anr.2024.04.004 38677471

[3] Deboever N Jones CM Yamashita K Ajani JA Hofstetter WL . Advances in diagnosis and management of cancer of the esophagus. BMJ. (2024) 385:e074962. doi: 10.1136/bmj-2023-074962 38830686

[4] Inoue J Morishita S Okayama T Suzuki K Tanaka T Nakano J . Impact of quality of life on mortality risk in patients with esophageal cancer: a systematic review and meta-analysis. Esophagus. (2024) 21(3):270–82. doi: 10.1007/s10388-024-01064-w 38772959

[5] Bandidwattanawong C . Multi-disciplinary management of esophageal carcinoma: current practices and future directions. Crit Rev Oncol Hematol. (2024) 197:104315. doi: 10.1016/j.critrevonc.2024.104315 38462149

[6] Guo W Li B Xu W Cheng C Qiu C Sam SK . Multi-omics and multi-VOIs to predict esophageal fistula in esophageal cancer patients treated with radiotherapy. J Cancer Res Clin Oncol. (2024) 150(2):39. doi: 10.1007/s00432-023-05520-5 38280037 PMC10821966

[7] Chen B Deng M Yang C Dragomir MP Zhao L Bai K . High incidence of esophageal fistula on patients with clinical T4b esophageal squamous cell carcinoma who received chemoradiotherapy: a retrospective analysis. Radiother Oncol. (2021) 158:191–199. doi: 10.1016/j.radonc.2021.02.031 33667583

[8] Qu J Wang Z Zhang H Lu Y Jia Z Lu S . How to update esophageal masses imaging using literature review (MRI and CT features). Insights Imaging. (2024) 15(1):169. doi: 10.1186/s13244-024-01754-0 38971944 PMC11227487

[9] Tang T Cui Y Lu C Li H Zhou J Zhang X . Evaluating performance of a deep learning multilabel segmentation model to quantify acute and chronic brain lesions at MRI after stroke and predict prognosis. Radiol Artif Intell. (2025) 7(3):e240072. doi: 10.1148/ryai.240072 40136026

[10] Zhang Y Cheng X Luo X Sun R Huang X Liu L . Prediction of esophageal fistula in radiotherapy/chemoradiotherapy for patients with advanced esophageal cancer by a clinical-deep learning radiomics model: prediction of esophageal fistula in radiotherapy/chemoradiotherapy patients. BMC Med Imaging. (2024) 24(1):313. doi: 10.1186/s12880-024-01473-4 39558242 PMC11571992

[11] Shi YJ Liu C Wei YY Li XT Shen L Lu ZH . Quantitative CT analysis to predict esophageal fistula in patients with advanced esophageal cancer treated by chemotherapy or chemoradiotherapy. Cancer Imaging. (2022) 22(1):62. doi: 10.1186/s40644-022-00490-2 36333763 PMC9636691

[12] Zhou Y Jiang F Cheng F Li J . Detecting representative characteristics of different genders using intraoral photographs: a deep learning model with interpretation of gradient-weighted class activation mapping. BMC Oral Health. (2023) 23(1):327. doi: 10.1186/s12903-023-03033-8 37231478 PMC10214706

[13] Dabass M Vashisth S Vig R . A convolution neural network with multi-level convolutional and attention learning for classification of cancer grades and tissue structures in colon histopathological images. Comput Biol Med. (2022) 147:105680. doi: 10.1016/j.compbiomed.2022.105680 35671654

[14] Iqbal S Qureshi AN Alhussein M Aurangzeb K Anwar MS . AD-CAM: enhancing interpretability of convolutional neural networks with a lightweight framework - from black box to glass box. IEEE J Biomed Health Inform. (2023) 28(1):514–25. doi: 10.1109/JBHI.2023.3329231 37910403

[15] Jiang PT Zhang CB Hou Q Cheng MM Wei Y . LayerCAM: exploring hierarchical class activation maps for localization. IEEE Trans Image Process. (2021) 30:5875–88. doi: 10.1109/TIP.2021.3089943 34156941

[16] Ikram A Imran A . ResViT FusionNet model: an explainable AI-driven approach for automated grading of diabetic retinopathy in retinal images. Comput Biol Med. (2025) 186:109656. doi: 10.1016/j.compbiomed.2025.109656 39823821

[17] Day TG Matthew J Budd SF Venturini L Wright R Farruggia A . Interaction between clinicians and artificial intelligence to detect fetal atrioventricular septal defects on ultrasound: how can we optimize collaborative performance?. Ultrasound Obstet Gynecol. (2024) 64(1):28–35. doi: 10.1002/uog.27577 38197584

[18] Jiang H Du Y Lu Z Wang B Zhao Y Wang R . Radiomics incorporating deep features for predicting Parkinson's disease in (123)I-Ioflupane SPECT. EJNMMI Phys. (2024) 11(1):60. doi: 10.1186/s40658-024-00651-1 38985382 PMC11236833

[19] Marcinkiewicz AM Buchwald M Shanbhag A Bednarski BP Killekar A Miller RJH . AI for multistructure incidental findings and mortality prediction at chest CT in lung cancer screening. Radiology. (2024) 312(3):e240541. doi: 10.1148/radiol.240541 39287522 PMC11427857

[20] Le NQK . Hematoma expansion prediction: still navigating the intersection of deep learning and radiomics. Eur Radiol. (2024) 34(5):2905–2907. doi: 10.1007/s00330-024-10586-x 38252277

[21] Le VH Minh TNT Kha QH Le NQK . A transfer learning approach on MRI-based radiomics signature for overall survival prediction of low-grade and high-grade gliomas. Med Biol Eng Comput. (2023) 61(10):2699–2712. doi: 10.1007/s11517-023-02875-2 37432527

[22] Li Z Gong J Shi L Li J Yang Z Chai G . Clinical-radiomics nomogram for the risk prediction of esophageal fistula in patients with esophageal squamous cell carcinoma treated with intensity-modulated radiation therapy or volumetric-modulated arc therapy. J Thorac Dis. (2024) 16(3):2032–2048. doi: 10.21037/jtd-24-191 38617757 PMC11009608

[23] Sato H Nishikawa K Hamakawa T Kusunoki C Miyake M Miyamoto A . Evaluating neoadjuvant chemotherapy for lower esophageal squamous cell carcinoma by measuring esophageal wall thickness. Anticancer Res. (2022) 42(11):5655–5662. doi: 10.21873/anticanres.16074 36288872

[24] Zhu C Sun W Chen C Qiu Q Wang S Song Y . Prediction of malignant esophageal fistula in esophageal cancer using a radiomics-clinical nomogram. Eur J Med Res. (2024) 29(1):217. doi: 10.1186/s40001-024-01746-2 38570887 PMC10993504

[25] Yu Z Xu C Song B Zhang S Chen C Li C . Tissue fibrosis induced by radiotherapy: current understanding of the molecular mechanisms, diagnosis and therapeutic advances. J Transl Med. (2023) 21(1):708. doi: 10.1186/s12967-023-04554-0 37814303 PMC10563272

[26] Venkatesulu BP Mahadevan LS Aliru ML Yang X Bodd MH Singh PK . Radiation-induced endothelial vascular injury: a review of possible mechanisms. JACC Basic Transl Sci. (2018) 3(4):563–572. doi: 10.1016/j.jacbts.2018.01.014 30175280 PMC6115704

[27] Danielpour D . Advances and challenges in targeting TGF-beta isoforms for therapeutic intervention of cancer: a mechanism-based perspective. Pharmaceuticals (Basel). (2024) 17(4):533. doi: 10.3390/ph17040533 38675493 PMC11054419

[28] Parichatikanond W Luangmonkong T Mangmool S Kurose H . Therapeutic targets for the treatment of cardiac fibrosis and cancer: focusing on TGF-beta signaling. Front Cardiovasc Med. (2020) 7:34. doi: 10.3389/fcvm.2020.00034 32211422 PMC7075814

[29] Erin N Akman M . Effects of in-vitro modulation of TRPV1 activity on immune response of mice bearing metastatic breast carcinoma: enhanced inflammatory response may hinder therapeutic potentials of TRPV1 agonists. Life Sci. (2021) 287:120115. doi: 10.1016/j.lfs.2021.120115 34740578

[30] Adapala RK Katari V Teegala LR Thodeti S Paruchuri S Thodeti CK . TRPV4 mechanotransduction in fibrosis. Cells. (2021) 10(11):3053. doi: 10.3390/cells10113053 34831281 PMC8619244

[31] Miida S Arao Y Takeda N Goto S Kojima Y Kimura N . A rare cause of esophageal stenosis: compression due to a thoracic osteophyte. DEN Open. (2024) 4(1):e260. doi: 10.1002/deo2.260 37409322 PMC10318124

[32] Halder S Yamasaki J Acharya S Kou W Elisha G Carlson DA . Virtual disease landscape using mechanics-informed machine learning: application to esophageal disorders. Artif Intell Med. (2022) 134:102435. doi: 10.1016/j.artmed.2022.102435 36462900

[33] Cellina M Pirovano M Ciocca M Gibelli D Floridi C Oliva G . Radiomic analysis of the optic nerve at the first episode of acute optic neuritis: an indicator of optic nerve pathology and a predictor of visual recovery?. Radiol Med. (2021) 126(5):698–706. doi: 10.1007/s11547-020-01318-4 33392980

[34] Dash AS Breighner R Gonzalez FQ Blumberg O Koff MF Billings E . Individuals with heterogenous trabecular bone texture by clinical magnetic resonance imaging have lower bone strength and stiffness by quantitative computed tomography-based finite element analysis. J Bone Miner Res. (2025) 40(3):339–347. doi: 10.1093/jbmr/zjae207 39731439 PMC13463277

[35] Wu Z Ge R Wen M Liu G Chen Y Zhang P . ELNet: automatic classification and segmentation for esophageal lesions using convolutional neural network. Med Image Anal. (2021) 67:101838. doi: 10.1016/j.media.2020.101838 33129148

[36] Yang M Ma J Zhang C Zhang L Xu J Liu S . Multimodal data deep learning method for predicting symptomatic pneumonitis caused by lung cancer radiotherapy combined with immunotherapy. Front Immunol. (2024) 15:1492399. doi: 10.3389/fimmu.2024.1492399 39845959 PMC11751032

