# Supplementary figures and images for: Spatial associations between esophageal lesions and surrounding tissues in esophageal fistula: a CAM-guided radiomics study
Source: Front Oncol. 2026 Jul 15;16:1825313. doi: 10.3389/fonc.2026.1825313 (PMC13414950; doi:10.3389/fonc.2026.1825313)

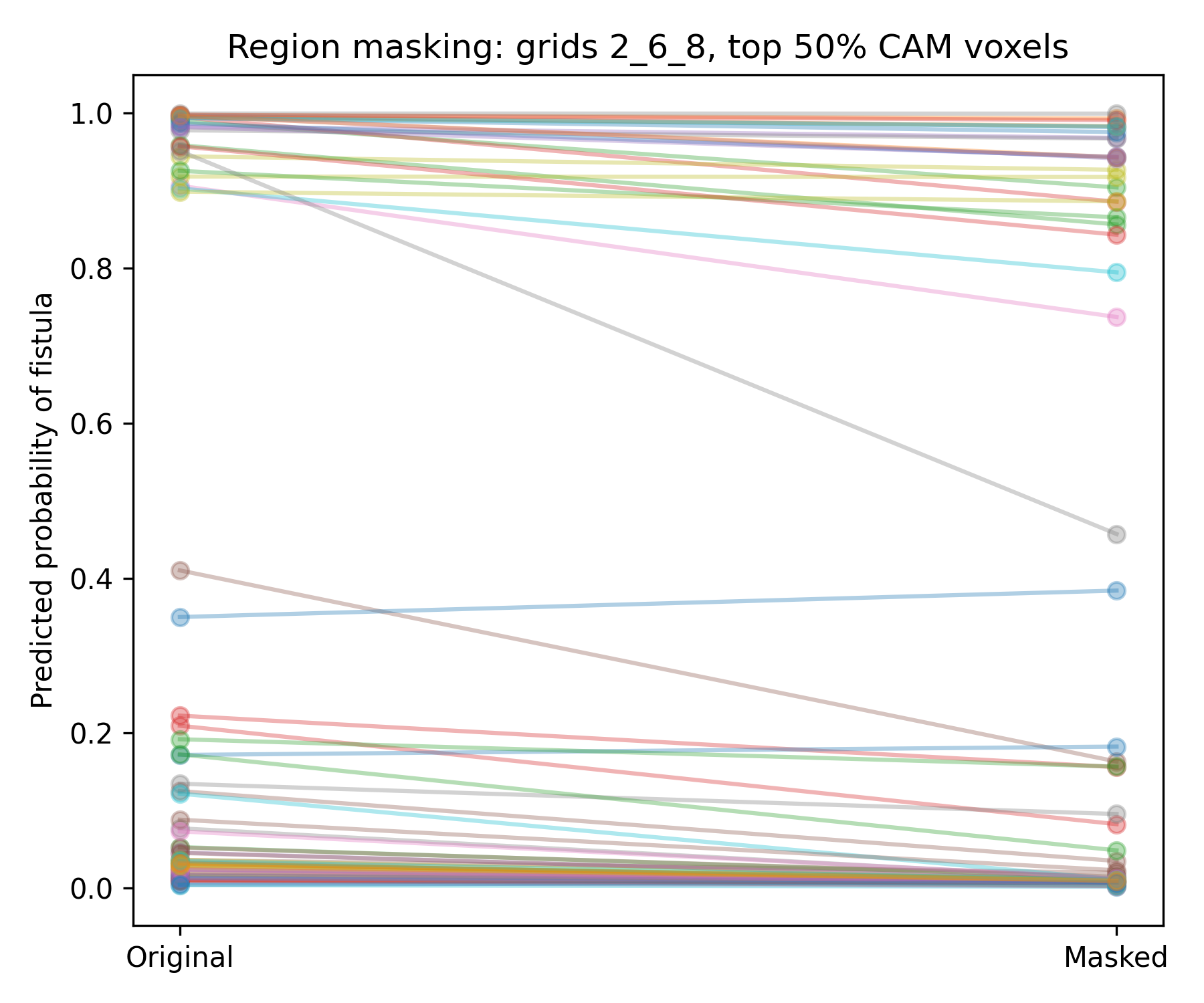

Supplement: Supplementary file 1 [file Image1.png]

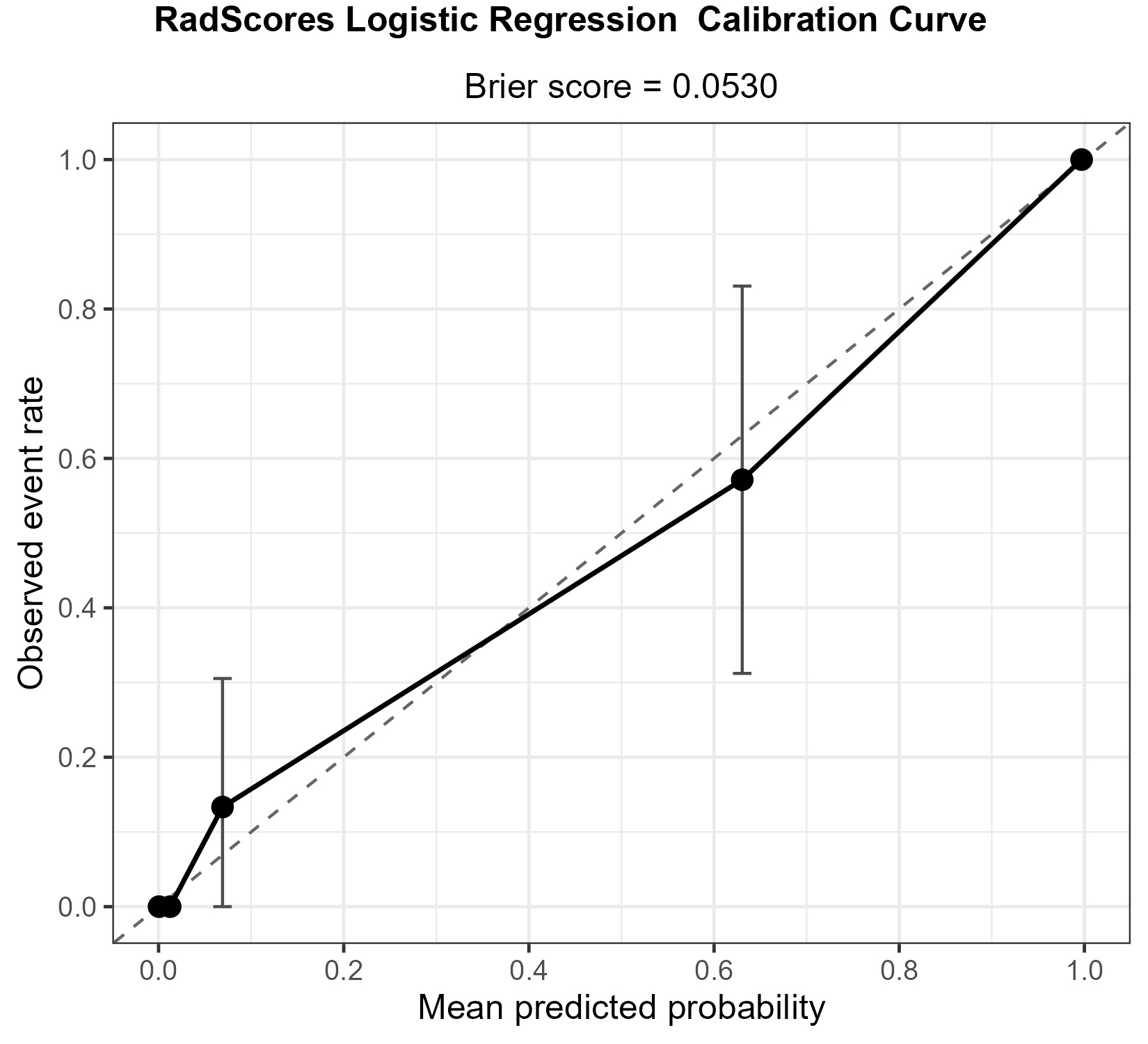

Supplement: Supplementary file 2 [file Image2.png]
