# Supplementary material for: Spatial associations between esophageal lesions and surrounding tissues in esophageal fistula: a CAM-guided radiomics study
Source: Front Oncol. 2026 Jul 15;16:1825313. doi: 10.3389/fonc.2026.1825313 (PMC13414950; doi:10.3389/fonc.2026.1825313)
Supplement: Supplementary file 3 [file Table1.docx]

| Folds | Accuracy | Precision | Recall | F1 | AUC | Sensitivity | Specificity | PPV | NPV |
| --- | --- | --- | --- | --- | --- | --- | --- | --- | --- |
| 1 | 0.800 | 0.818 | 0.900 | 0.857 | 0.860 | 0.900 | 0.600 | 0.818 | 0.750 |
| 2 | 0.933 | 0.909 | 1.000 | 0.952 | 0.800 | 1.000 | 0.800 | 0.909 | 1.000 |
| 3 | 0.667 | 0.727 | 0.800 | 0.762 | 0.760 | 0.800 | 0.400 | 0.727 | 0.500 |
| 4 | 0.857 | 1.000 | 0.778 | 0.875 | 0.933 | 0.778 | 1.000 | 1.000 | 0.714 |
| 5 | 0.786 | 0.800 | 0.889 | 0.842 | 0.889 | 0.889 | 0.600 | 0.800 | 0.750 |
| Mean | 0.809 | 0.851 | 0.873 | 0.858 | 0.848 | 0.873 | 0.680 | 0.851 | 0.743 |

**Table S1 Five-Fold Cross-Validation Performance of the Esophageal Fistula Prediction**
